# A Novel Gamma Distributed Random Variable (RV) Generation Method for Clutter Simulation with Non-Integral Shape Parameters

**DOI:** 10.3390/s20040955

**Published:** 2020-02-11

**Authors:** Shichao Chen, Feng Luo, Chong Hu

**Affiliations:** National Laboratory of Radar Signal Processing, Xidian University, Xi’an 710071, China; scchen0115@163.com (S.C.); hake_hc@163.com (C.H.)

**Keywords:** clutter simulation, Gamma distribution, compound Gaussian distributed model

## Abstract

Sea clutter simulation is a well-known research endeavour in radar detector analysis and design, and many approaches to it have been proposed in recent years, among which zero memory non-linear (ZMNL) and spherically invariant random process (SIRP) are the most two widely used methods for compound Gaussian distribution. However, the shape parameter of the compound Gaussian clutter model cannot be a non-integer nor non-semi-integer in the ZMNL method, and the computational complexity of the SIRP method is very high because of the complex non-linear operation. Although some improved methods have been proposed to solve the problem, the fitting degree of these methods is not high because of the introduction of Beta distribution. To overcome these disadvantages, a novel Gamma distributed random variable (RV) generation method for clutter simulation is proposed in this paper. In our method, Gamma RV with non-integral or non-semi-integral shape parameters is generated directly by multiplying an integral-shape-parameter Gamma RV with a Beta RV whose parameters are larger than 0.5, thus avoiding the deviation of simulation of Beta RV. A large number of simulation experimental results show that the proposed method not only can be used in the clutter simulation with a non-integer or non-semi-integer shape parameter value, but also has higher fitting degree than the existing methods.

## 1. Introduction

Clutter is one of the main factors restricting radar target detection and tracking performance. The research on sea clutter is of great significance for radar detection and system design [1,2]. However, sea clutter data in the actual collection usually do not conform to the specific precise model. Therefore, the method of clutter simulation based on a specific parameter model is particularly important in radar system analysis and performance verification [3,4]. Because of the complexity and rapidity of the sea condition, the research of sea surface remote sensing also presents the development trend from monostatic radar to multistatic radar [5,6], from conventional band radar to microwave radar [7], and from traditional radar to multi-element radar [8,9]. Compound Gaussian distribution, such as K distribution and Pareto distribution, which can better describe many kinds of clutter with high resolution and low grazing angle, has becoming a crucial statistical model for clutter simulation [10,11,12].

In order to simulate the compound Gaussian clutter accurately, many scholars at home and abroad have conducted research on coherent and incoherent clutter models. A correlated coherent K-distributed clutter based on the spherically invariant random process (SIRP) is presented in [11]. The SIRP can control the power spectrum and amplitude independently, but it is constrained by the sequence order and autocorrelation function (ACF), and it is difficult to form a fast algorithm because of its large computation. A method based on zero memory non-linear (ZMNL) is proposed in [12]. The ZMNL is suitable for non-coherent clutter model, and the principle of it is simple. However, the main drawback of ZMNL is that the shape parameter of the clutter model must be an integer or semi-integer. If the shape parameter does not satisfy the requirement, the ZMNL method will lead to clutter simulation error. What’s worse, the ZMNL cannot control the power spectrum and amplitude independently. With the principle that each quadrature component of K-distributed clutter can be modelled, exactly or approximately, by a weighted sum of products of two independent Gaussian variables in [13], a new method for modelling and simulation of correlated K-distributed clutter is proposed in [14]; the new method can simulate the clutter with arbitrary and specified power spectrum, and is a much more simple calculation compared with the traditional ZMNL and SIRP, which makes it easier to realize both in software and hardware. Nevertheless, an approximate approach is made for the simulation when the shape parameter is non-integer or non-semi-integer. Besides, a method of simulation of coherent compound Gaussian clutter based on memorials non-linear transformation (MNLT) is proposed in [15]. Through the generation of arbitrary correlated Gamma random variable (RV) by MNLT, this method can simulate clutter specifically. However, similar to SIRP method, the computational complexity of MNLT is high due to nonlinear computation.

To solve this problem, the additive property of Gamma RV is used to improve the ZMNL and SIRP methods in [16,17], in which the shape parameter of the compound Gaussian distribution is the same as the Gamma distribution. The shape parameter $v_{1}$ is divided into two parts: one is the integral or semi-integral named as $v_{1}$, and the other is the non-integral or non-semi-integral named as $v_{2}$($0<v_{2}<0.5$). The specific Gamma RV with $v_{2}$ is produced by multiplying a Beta RV with another Gamma RV of integer or semi-integer shape parameter in [16], while the specific Gamma RV with $v_{2}$ is obtained through the product of Beta RV with exponential distribution RV in [17]. Then, the Gamma RV is applied to the simulation of K distributed clutter. The improved Gamma RV generation method has been applied in clutter simulation based on SIRP in [17], which avoids the calculation of non-linear equations. Although the above approaches solve the problem of the approximation of shape parameter, there is a certain deviation between its statistical histogram and theoretical Probability Density Function (PDF) because the simulation of Beta RV is deviated when the parameter is small, which will affect the obtained Gamma RV, and lead to the final clutter error.

To overcome this disadvantage, an improved Gamma distributed RV generation method is proposed in this paper. In the proposed method, the Gamma RV with non-integral or non-semi-integral shape parameters is generated directly by multiplying an integral-shape-parameter Gamma RV with a Beta RV whose parameters are larger than 0.5, thus avoiding the deviation of simulation of Beta RV. Compared with the existing clutter simulation methods, the proposed improved method not only can be used in the clutter simulation with a non-integer or non-semi-integer shape parameter values, but also has higher fitting degree than the existing methods. A large number of simulation experiments verify the effectiveness of our method.

## 2. Compound Gaussian Distribution

The compound Gaussian distributed model consists of two components, texture component and speckle component. As a slowly varying component with long time correlation, texture component reflects the average level of backward scattering in spatial variation associated with the large area structure of the sea surface. For K distributed clutter, the texture component is a Gamma distributed RV. For Pareto distributed clutter, it is an inverse Gamma distributed RV. Unlike the texture component, a speckle component is a rapidly varying component obeying Rayleigh distribution. Therefore, we can write the PDF of compound Gaussian distributed model as:

(1) $$f_{N}(z)=\int_{0}^{\infty }f_{Z/X}(z|x,N)f_{X}(x)dx$$

where z is the intensity of clutter signal, x is the intensity of signal for texture component, *N* is the observational frequency or the accumulation number of pulse, p(z|x,N) is the speckle component, $p_{x}(x)$ is the texture component.

For multi-look compound Gaussian distributed clutter, its speckle component obeys Gamma distribution. The shape parameter is *N* for Gamma distribution when the number of the independent observation is *N* [5]. The PDF of it can be written as:

(2) $$f_{Z|X}(z|x,N)=\frac{z^{N-1}}{x^{N}\Gamma (N)}\exp(-\frac{z}{x})$$

where Γ(⋅) is the Gamma function. When N=1, speckle component obeys exponential distribution.

For *K* distribution, its texture component obeys Gamma distribution, and the PDF of it is:

(3) $$f_{X}(x)=G(x;\alpha ,\beta )=\frac{\beta^{\alpha }}{\Gamma (\alpha )}x^{\alpha -1}\exp\left(-\beta x\right)$$

where x≥β, the shape parameter α>0 determines the overall shape of the distribution. The scale parameter β>0 determines where the distribution support begins.

For Pareto distribution, the texture component of it obeys inverse Gamma distribution, and the PDF can be written as:

(4) $$f_{X}(x)=\mathrm{IG}(x;\alpha ,\beta )=\frac{\beta^{\alpha }}{\Gamma (\alpha )}x^{-\alpha -1}\exp\left(-\frac{\beta }{x}\right)$$

Therefore, it is required to generate Gamma RV first to generate *K*-distributed or Pareto-distributed clutter. The generation of inverse Gamma RV can be obtained by the reciprocal transformation of Gamma RV. Moreover, the fact that the parameters of *K* and Pareto distribution are the same as the Gamma and inverse Gamma’s is in evidence.

## 3. Problem Formulation

### 3.1. Irrandomicity of Parameter for Zero Memory Non-Linear (ZMNL)

**Figure 1:**
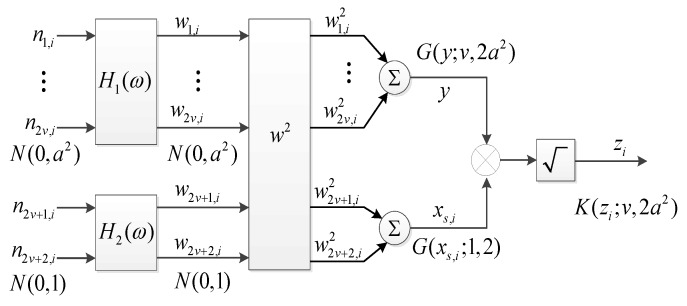
Flow diagram of zero memory non-linear (ZMNL) for generating correlated compound Gaussian-distributed clutter.

Utilizing the ZMNL method to generate sea clutter consists of two branches, one is to generate texture component y that can be obtained by the accumulation of 2v normal distributed RVs as $\left\{n_{1,i},\ldots ,n_{2v,i}\right\}$, let the shape parameter α=v, scale parameter $\beta =2a^{2}$ in Figure 1, where $y\sim G(y,\alpha ,\beta )=G(y,v,2a^{2})$; the other is to generate speckle component $x_{s,i}$, where $x_{s,i}\sim G(x_{s,i},1,2)$, it can be obtained by the sum of the squares of two exponential RVs $n_{2v+1,i}$ and $n_{2v+2,i}$, the specific process is shown in Figure 1.

Thus it can be seen that the shape parameter of clutter through the ZMNL method must be an integer or semi-integer from the process. If the shape parameter is non-integral or non-semi-integral, the ZMNL is improper.

For the clutter correlation, in order to make the simulated clutter not only satisfy the complex Gaussian distribution in amplitude (taking K-distribution as an example), but also satisfy the required Gaussian spectral characteristics in the power spectrum, the correlation coefficient of K-distribution clutter sequence can be controlled and changed by adjusting the filter coefficient $S_{ij}$:

(5) Sij=Λ2[F12(−1/2,−1/2;ν+1;rij2)2F1(−1/2,−1/2;ν+1;qij2)−1]ν+1−Λ2

where F21(α,β;γ;η) is the Gaussian hypergeometric distribution function, Λ=Γ(ν+3/2)Γ(3/2)Γ(ν+1), $r_{ij}$ and $q_{ij}$ is the coefficients of the filters. The above formulation has infinite combination solutions. In the actual simulation process, the correlation coefficient can be derived by the Fourier series expansion method, and then the combination of $r_{ij}$ and $q_{ij}$ can be substituted into the above formulation, so as to calculate the coefficients of the filters.

### 3.2. Limitation of Beta Random Variable (RV)

In [14], according to the additive property of the Gamma distributed shape parameter, Conte overcame the shortcoming of the traditional ZMNL. It is assumed that the compound Gaussian distributed model is related to a Gamma variable sequence with parameters as $y\sim G\left(y;v,2a^{2}\right)$.

(a) If v is an integer, then y is the sum of v exponential variable sequences with parameter v.

(b) If v is a semi-integer, then y is a chi-square variable sequence and hence can be easily generated as the square root of the sum of 2v squared independent standard Gaussian variable sequences.

(c) If v is neither an integer nor semi-integer, then divided the shape parameter v into an integral or semi-integral part $v_{1}$ and a non-integral or non-semi-integral part $v_{2}$ ($0<v_{2}<0.5$). A Gamma RV $y_{1}$ with shape parameter $v_{1}$ is generated by adding up $2v_{1}$ independent and identically distributed (i.i.d) squared normal RVs while the other Gamma RV $y_{2}$ with shape parameter $v_{2}$ is generated by multiplying a Beta RV as $\beta \sim \mathrm{Be}(\beta ;v_{2},1-v_{2})$ and an exponential RV with unit parameter. That is:

(6) $$y_{2}=\eta \beta /2a^{2}\sim G(y_{2};v_{2},2a^{2})$$

where the PDF of Beta distribution with parameter (λ,χ) is:

(7) $$\begin{array}{l} Beta(\beta ;\lambda ,\chi )=\frac{\Gamma (\lambda +\chi )}{\Gamma (\lambda )\Gamma (\chi )}\beta^{\lambda -1}{\left(1-\beta \right)}^{\chi -1},\\ 0\leq \beta \leq 1,\lambda >0,\chi >0 \end{array}$$

The PDF of Gamma distribution with parameter (α,β) is:

(8) $$G(y;\alpha ,\beta )=\frac{1}{\beta^{\alpha }\Gamma (\alpha )}y^{\alpha -1}e^{-\frac{y}{\beta }},\;y>0$$

Then $y=y_{1}+y_{2}$ is a Gamma RV with shape parameter $v_{1}+v_{2}$. That is:

(9) $$y=y_{1}+y_{2}\sim G\left(y;v_{1}+v_{2},2a^{2}\right)$$

The RV β~Beta(β;λ,χ) is usually generated by the following general rejection method [14,16,17]:

(a) Generate two uniform RVs with unit parameter, $U_{1}$ and $U_{2}$;

(b) Evaluate $Y_{1}=U_{1}^{1/\lambda }$ and $Y_{2}=U_{2}^{1/\chi }$;

(c) If $Y_{1}+Y_{2}>1$, then go to Step (a), otherwise deliver:

(10) $$\beta =\frac{Y_{1}}{Y_{1}+Y_{2}}$$

**Figure 2:**
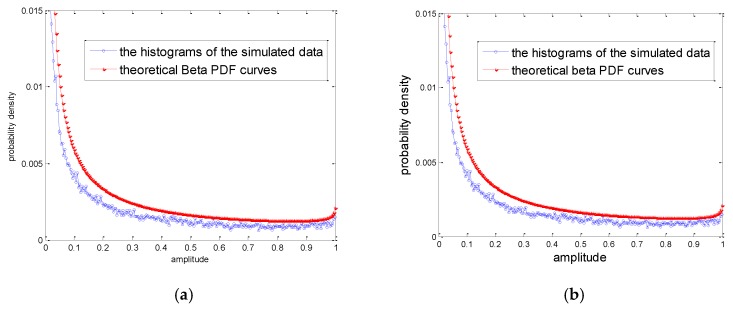
The histograms of the simulated data and the corresponding theoretical Beta probability density function (PDF) curves. (**a**) λ=0.15 χ=0.85; (**b**) λ=1.15 χ=1.85.

However, when λ<0.5 or χ<0.5, the histogram of the simulated data deviates the theoretical Beta PDF curve in the small value interval as Figure 2a shows. Figure 2b is the histograms of the simulated data and the corresponding theoretical Beta PDF curves of λ>0.5. It is found that when λ>0.5, the histograms of the simulated data fit the theoretical PDF curves better.

Similar but different to the method of Conte, Wallace generated the Gamma RV with shape parameter $v_{2}$ by multiplying a Beta RV $\beta \sim \mathrm{Be}(\beta ;v_{2},2-v_{2})$ and a Gamma distributed RV [16]. However, both of them have the deviation with general rejection method due to $v_{2}<0.5$.

Inspired by the works of the predecessors, we come to the conclusion, of which the methods Conte and Wallace are the special cases, that a Gamma RV with shape parameter v can be obtained by multiplying a Beta RV β~Beta(β;v,n−v) and a Gamma RV with shape parameter n, which is proved in this paper mathematically.

## 4. Proposed Method for Clutter Simulation

### 4.1. Simulation of Gamma RV

The proposed method is to generate the Gamma RV with non-integral or non-semi-integral shape parameters by directly multiplying an integral-shape-parameter Gamma RV and a Beta RV whose parameters are larger than 0.5. Hence, the proposed method not only solves the problem that the value of shape parameter cannot be non-integral or non-semi-integral, but also does not need to divide the parameter v into $v_{1}$ and $v_{2}$, and thus the process of simulation is more simple.

The proposed method is stated as: If $g\sim G(g;k+m,2a^{2})$ and β~Be(β;k+l,m−l), then $f_{y,z}=g\cdot \beta \sim G(y;k+l,2a^{2})$, where k and m are two positive integers and 0<l<1, thus k+l is a non-integer. Obviously, the method in [16] is a special situation of ours when k=0, m=1 and the method in [17] is the special when k=0, m=2.

The conclusion above is proved as follows: g and β are independent mutually, so the joint PDF of the 2-dimensional RV(g,β) is:

(11) $$f_{g,\beta }(g,\beta )=\frac{1}{{\left(2a^{2}\right)}^{k+m}\Gamma (k+l)\Gamma (m-l)}\beta^{k+l-1}{\left(1-\beta \right)}^{m-l-1}g^{k+m-1}e^{-\frac{g}{2a^{2}}}$$

Step 1. To deduce the PDF of y firstly. We make the transform of z=g and y=gβ≤z, and compute the Jacobian of this transform:

(12) $$J=\left|\begin{array}{cc} \frac{\partial g}{\partial y} & \frac{\partial \beta }{\partial y}\\ \frac{\partial g}{\partial z} & \frac{\partial \beta }{\partial z} \end{array}\right|=\left|\begin{array}{cc} 0 & \frac{1}{z}\\ 1 & -\frac{y}{z^{2}} \end{array}\right|=-\frac{1}{z}$$

Step 2. Then the PDF of the 2-dimensional RV (y,z) can be obtained as:

(13) $$\begin{array}{cc} f_{y,z}(y,z) & =f_{g,\beta }(z,\frac{y}{z})\left|J\right|\\ & =\frac{1}{{\left(2a^{2}\right)}^{k+m}\Gamma (k+l)\Gamma (m-l)}y^{k+l-1}{\left(z-y\right)}^{m-l-1}e^{-\frac{z}{2a^{2}}} \end{array}$$

Step 3. By integrating on z from y to +∞, the PDF of y is calculated as:

(14) $$\begin{array}{cc} f_{y}(y) & =\int_{y}^{+\infty }f_{y,z}(y,z)dz\\ & =\frac{1}{{\left(2a^{2}\right)}^{k+m}\Gamma (k+l)\Gamma (m-l)}y^{k+l-1}\int_{y}^{+\infty }{\left(z-y\right)}^{m-l-1}e^{-\frac{z}{2a^{2}}}dz \end{array}$$

Step 4. Finally, supposing t=z−y, Equation (13) can be written as:

(15) $$\begin{array}{cc} f_{y}(y) & =\frac{1}{\left(2a^{2}{)}^{k+m}\Gamma (k+l)\Gamma (m-l\right)}y^{k+l-1}e^{-\frac{y}{2a^{2}}}\int_{0}^{+\infty }t^{m-l-1}e^{-\frac{t}{2a^{2}}}dt\\ & =G(y;k+l,2a^{2}) \end{array}$$

Since we have generated a Gamma RV with a non-integral shape parameter, we can get an inverse Gamma RV with the same parameter from the reciprocal of the Gamma RV.

### 4.2. Simulation of Compound Gaussian Distributed Clutter

**Figure 3:**
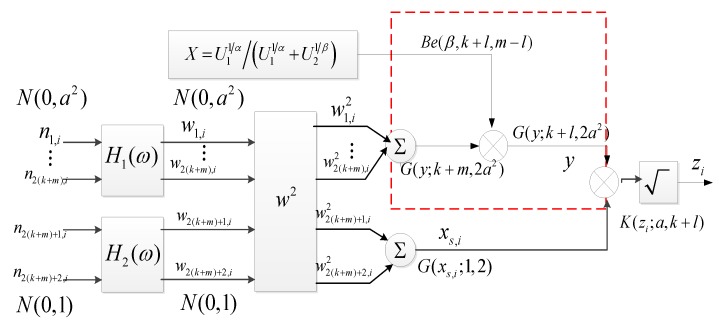
Flow diagram of the proposed improved method for generating correlated K-distributed clutter based on ZMNL.

If the RVs $x_{s,i}\sim G(x_{s,i};1,2)$ and $y\sim G(y;k+l,2a^{2})$ are independent, then $z_{i}=\sqrt{x_{s,i}y}\sim G(z_{i};k+l,2a^{2})$ is a K distributed RV. The specific process of the proposed improvement of ZMNL is shown in dotted frame in Figure 3. Although the computation cost of the Conte’s method and the proposed are both $O(n^{2})$ when the length of clutter sequence is n, the proposed method removes the branch to generate the exponential RV, which can save some hardware resources in the simulation project.

In the SIRP, the compound Gaussian distribution is treated as the modulation result of a non-negative RV, which square follows the Gamma distribution, to a complex Gaussian random sequence through nonlinear transform. In [17], the process of solving nonlinear equations in traditional SIRP is replaced by the method the proposed for Gamma RV generation to avoid the inefficiency brought by non-linear equations, which can transform integral arithmetic into simple addition and multiplication. In the same way, our method can also be extended to any other clutter simulation involved Gamma RV generation, such as SIRP and MNLT, to reduce the amount of computation to some extent. Compared with the traditional SIRP method, if the dichotomy method is used to solve the non-linear equation, the query interval is set as $\left[s_{1},s_{2}\right]$, and the error accuracy is assumed to be ε, the length of the composite Gaussian distribution sequence to be generated is L, and the number of low-pass filters is M, the operation amount is O(ML), and then it needs to solve the *L*-th non-linear equation, and each solution of the equation needs to be carried out $\log_{2}\left(\left(s_{2}-s_{1}\right)/\varepsilon \right)$ times integral calculation. However, the operation amount of the improved method is just $O\left(v_{1}ML\right)$. The upper limit of shape parameter in practice is 20, and the calculation of integral is much more than that of addition or multiplication [17].

## 5. Simulation Results

This section is not mandatory, but can be added to the manuscript if the discussion is unusually long or complex.

The simulation parameters are listed in Table 1.

**Figure 4:**
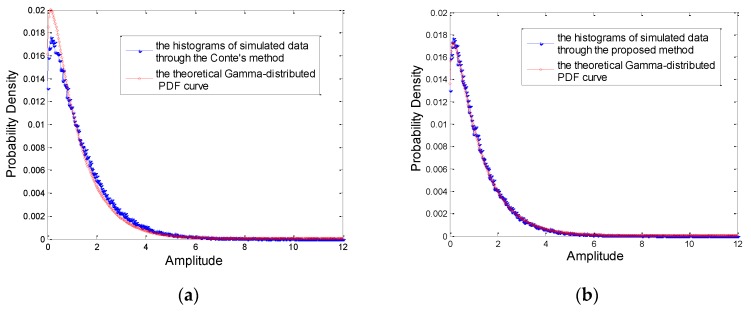

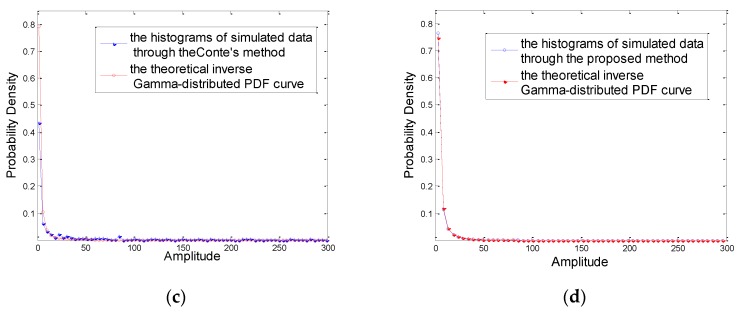
Theoretical Gamma and inverse Gamma PDFs and averaged histogram of the simulated data. (**a**) the simulated data of Gamma distribution through Conte’s method. (**b**) the simulated data of Gamma distribution through the proposed method. (**c**) the simulated data of inverse Gamma distribution through Conte’s method. (**d**) the simulated data of inverse Gamma distribution through the proposed method.

Firstly, we compare the proposed method with Conte’s method. In the experiment, the shape parameter is set 1.15 to compare the fitting degree conveniently. We set n=1, m=3 and l=0.15 in the proposed method while v divided into $v_{1}=1$ and $v_{2}=0.15$ in Conte’s method. The Gamma distributed RV is generated through Conte’s method and the proposed method respectively. With the average of data simulated 1000 times, we compare the histograms of both simulated data with the theoretical PDF curve shown in Figure 4. Figure 4a shows the average histogram of simulated data through Conte’s method, while Figure 4b is the average histogram of simulated data by the proposed method. Figure 4c,d are the results of inverse Gamma distribution. It is apparent that the fitting degree of the proposed method between the average histogram of simulated data and the theoretical PDF curve is higher than that of Conte’s method.

**Figure 5:**
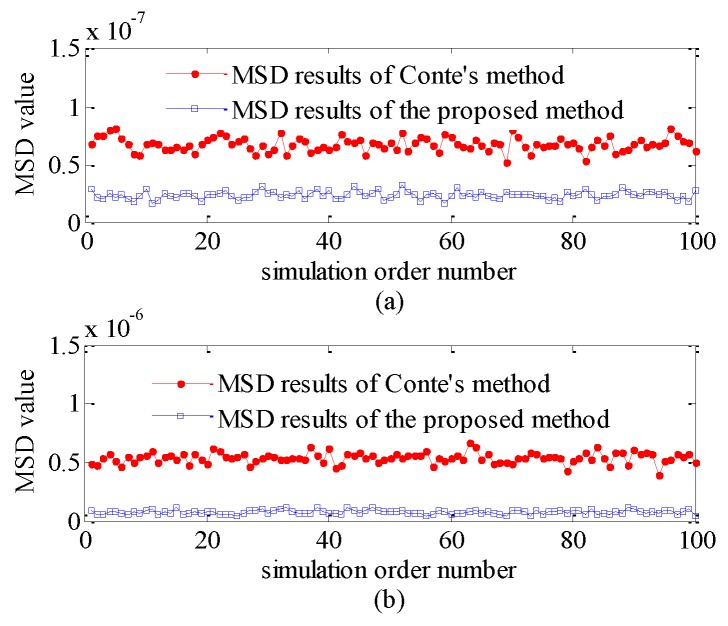
Mean squared difference (MSD) results of goodness of fit when utilizing different methods. (**a**) Gamma distribution (**b**) Inverse Gamma distribution.

To illustrate the performance of the proposed method further, the mean squared difference (MSD) technique is used to test the goodness-of-fit of the simulated data; simulation was done 100 times and a MSD value was obtained in each time. Figure 5 shows the MSD results with the number of simulation order for different methods when the clutter distribution is Gamma and inverse Gamma distributions. Figure 5a is the result of Gamma-simulated data and Figure 5b is the result of inverse Gamma simulated data. In Figure 5a,b, the red line is the MSD results of Conte’s method while the blue is the MSD results of the proposed method. It is obvious that the MSD values of the proposed method are lower than Conte’s method whether the clutter distribution is Gamma distribution or inverse Gamma distribution. Therefore, the fitting degree of the proposed method between the generated data and the theoretical PDF curve is higher than that of Conte’s method.

Next, we compare the performance of the proposed method with the existing method in [14]. In the existing method, the shape parameter v is divided into two parts: one is the integral or semi-integral named as $v_{1}$, and the other is the non-integral or non-semi-integral named as $v_{2}$($0<v_{2}<0.5$). The problem of the existing method is that $v_{2}$ will lead to deviation because of the properties of Beta function. Therefore, a novel method is proposed to solve this problem. The proposed method is stated as: if $g\sim G(g;k+m,2a^{2})$ and β~Be(β;k+l,m−l), where k and m are two positive integers, 0<l<1, G(⋅) is Gamma distribution, and Be(⋅) is Beta distribution. Then $f_{y,z}=g\cdot \beta \sim G(y;k+l,2a^{2})$, where, thus k+l is a non-integer.

In order to analyze the exhaustive performance of our method, MSD test statistics of Gamma distribution for the proposed method and the existing method is given based on the different value of the shape parameter. The shape parameter is set as follows: the non-integer part is set form 0.1 to 0.5 with the interval of 0.1, and the integer part is set as 0 and 1 respectively. We carry out multiple Monte Carlo experiments on the existing method and the improved method, and give the average value of MSD test statistic based on 100 simulation results. It is obvious from Table 2 that the MSD test statistic value of the proposed method is much smaller than that of the existing method, which demonstrate the effectiveness of the proposed method. Moreover, the proposed method can not only solve the problem that the value of shape parameter cannot be non-integral or non-semi-integral, but also greatly simplify the process of simulation.

**Figure 6:**
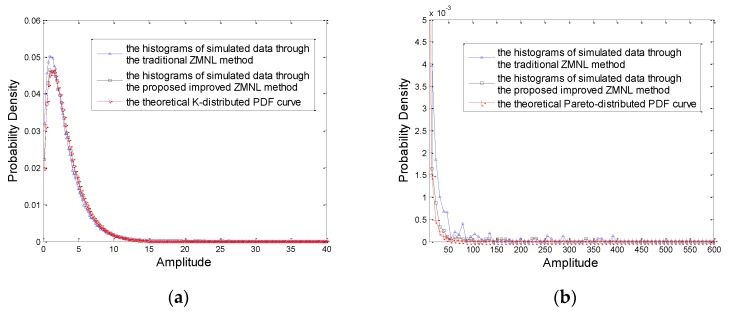
Theoretical PDF and averaged histogram of the simulated data generated by the traditional ZMNL and the proposed improved ZMNL methods. (**a**) K distribution; (**b**) Pareto distribution.

Then, we compare the performance of the proposed method with the traditional ZMNL method. The correlated K distributed and Pareto distributed RVs are generated by the traditional ZMNL method and the improved ZMNL method proposed in this paper, respectively. Since the value of the shape parameter of ZMNL can just be the integral or semi-integral value, the value of the shape parameter is set as 1 in this experiment. The histograms of the simulated data under K distribution and Pareto distribution are obtained with the average of 1000 times simulated data, which are shown in Figure 6a,b respectively. It can be seen from Figure 6 that the fitting degree of the proposed method is higher than the traditional ZMNL whether the clutter distribution is K distribution or Pareto distribution, especially when the value of amplitude is small.

**Figure 7:**
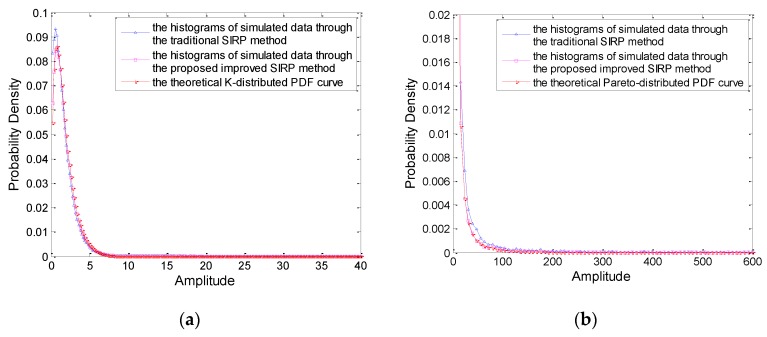
Theoretical PDF and averaged histogram of the simulated data generated by the traditional SIRP and the proposed improved SIRP methods. (**a**) K distribution (**b**) Pareto distribution.

Finally, we compare the performance of the proposed method with the traditional SIRP method. The correlated K-distributed and Pareto-distributed RVs are generated by the traditional SIRP method and the improved SIRP method proposed in this paper, respectively. The histograms of the simulated data under K distribution and Pareto distribution are obtained with the average of 1000 times simulated data, which are shown in Figure 7a, b respectively. It can be seen from Figure 7 that the fitting degree of the proposed method is higher than the traditional SIRP whether the clutter distribution is K distribution or Pareto distribution, especially when the value of amplitude is small.

## 6. Conclusions

A novel Gamma-distributed random variable (RV) generation method for clutter simulation with non-integral or non-semi-integral shape parameter is proposed in this paper. In the proposed method, the RV is generated directly by multiplying an integral-shape-parameter Gamma RV and a Beta RV whose parameters are larger than 0.5. Compared with the existing clutter simulation methods, the proposed method can not only simulate correlated compound Gaussian-distributed radar clutter with non-integral or non-semi-integral shape parameter, but also can avoid the deviation from Beta distribution. Therefore, the fitting degree of the proposed method between the generated data and the theoretical PDF curve is higher than the existing methods. Moreover, the simulation flow diagram based on the proposed method removes the process of generating the exponential RV which saves more hardware resource. Besides, the proposed method can be applied to other clutter simulation methods for generating Gamma RV. A large number of simulation experimental results demonstrate the performance of the proposed method.

## Figures and Tables

**Table 1:** Parameters.

Parameter	Value
Sampling frequency	1000 Hz
Total simulation number	8192
Scale parameter	1.85
Shape parameter	1.15

**Table 2:** MSD test statistics of Gamma distribution (Magnitude: $10^{-8}$).

The Shape Parameter	The Existing Method [14]			The Proposed Method			
	$v_{1}$	$v_{2}$	MSD Test Statistics	k	m	l	MSD Test Statistics
0.1	0	0.1	50.9643	0	2	0.1	9.4249
0.2	0	0.2	107.9483	0	2	0.2	7.3960
0.3	0	0.3	191.6507	0	2	0.3	14.8721
0.4	0	0.4	323.9830	0	2	0.4	26.5204
0.5	0	0.5	470.4800	0	2	0.5	47.6862
1.1	1	0.1	31.2007	1	2	0.1	7.2830
1.2	1	0.2	80.9828	1	2	0.2	6.7206
1.3	1	0.3	241.3822	1	2	0.3	14.6254
1.4	1	0.4	335.8412	1	2	0.4	21.9778
1.5	1	0.5	795.2914	1	2	0.5	45.3209
